# The impact of life events on NSSI among left-behind college students: the mediating role of PLEs and the moderating role of social support

**DOI:** 10.3389/fpsyg.2025.1573133

**Published:** 2025-08-18

**Authors:** Hongcai Wang, Yali Fu, Zihao Zeng, Lulu Lin, Qianyu Cheng, Juan Zhao, Yiqiu Hu

**Affiliations:** ^1^School of Educational Science, Hunan Normal University, Changsha, China; ^2^School of Electrical Information Engineering, Hunan Institute of Technology, Hengyang, China; ^3^Finance Department, Hunan Institute of Technology, Hengyang, Hunan, China; ^4^Mental Health Education Center, Central South University of Forestry and Technology, Changsha, Hunan, China; ^5^College of Computer Science, Guangdong University of Science and Technology, Dongguan, Guangdong, China; ^6^School of Elementary Education, Hunan First Normal University, Changsha, Hunan, China; ^7^Research Center for Mental Health Education of Hunan Province, Changsha, China; ^8^Cognition and Human Behavior Key Laboratory of Hunan Province, Changsha, China

**Keywords:** life events, NSSI, PLEs, social support, college students, left-behind experiences

## Abstract

This study investigated the relationship between life events and non-suicidal self-injury (NSSI) among Chinese college students with left-behind experiences, focusing on the mediating role of Psychotic-like experiences (PLEs) and the moderating role of social support in this relationship. A total of 7,577 students were surveyed using the Adolescent Self-Rating Life Events Checklist (ASLEC), 8 positive symptom items from the Community Assessment of Psychic Experiences (CAPE), the Perceived Social Support Scale (PSSS), and the Deliberate Self-Harm Inventory (DSHI). After excluding invalid questionnaires, 5,754 were retained, of which 2,772 college students had left-behind experiences. The results show that (1) The prevalence of NSSI among college students with left-behind experiences was 11.51%, which was higher than that of college students without left-behind experiences (9.66%); (2) PLEs partially mediated the effect of life events on NSSI; (3) Social support moderated the first-stage path, second-stage path, and direct path of the mediation model by attenuating the effects of life events on PLEs, PLEs on NSSI, and the direct effects of life events on NSSI. So, we conclude that, among college students with left-behind experiences, PLEs mediate the effect of life events on NSSI, while social support moderates the mediation model by influencing the first-stage path from life events to PLEs, the second-stage path from PLEs to NSSI, and the direct path from life events to NSSI.

## 1 Introduction

Since the 1980s, the massive migration of young and middle-aged rural adults to cities for work has led to a significant increase in the number of left-behind children in China. This demographic shift has profound implications for the psychological and social development of these children. By 2020, the total number of left-behind children in China reached 66.93 million, comprising 41.77 million rural left-behind children and 25.16 million urban left-behind children (National Bureau of Statistics, 2020). Left-behind experiences, often resulting from parent-child separation due to parental migration for work or grandparental caregiving, can impact an individual's mental health in adulthood. Moreover, the negative consequences of these experiences may be more severe than those associated with migration experiences (Wang and Liu, 2023). Some left-behind children go on to become college students, carrying with them the experiences of being left behind in their childhood as they reach adulthood. Prior research has demonstrated that college students with left-behind experiences have significantly lower levels of mental health compared to their counterparts without such experiences (Liu et al., 2021). Specifically, the prevalence of mental health issues among these students is 2.14 times higher, manifesting in symptoms such as somatization, obsessive-compulsive disorder, anxiety, and depression (Liu et al., 2021). Therefore, elucidating the occurrence and developmental mechanisms of non-suicidal self-injury (NSSI) among college students with left -behind experiences can yield valuable insights for the development of targeted prevention and intervention strategies.

There is a close relationship between life events and non-suicidal self-injury (NSSI). NSSI refers to behaviors in which individuals intentionally and repeatedly harm their own bodies in various ways, such as cutting or stabbing, without the intention of suicide (Nock, 2010). According to Nock's integrated theoretical model (Nock, 2009), NSSI functions as an effective mechanism for rapidly regulating negative emotional experiences and improving social situations. Additionally, life events can act as both distal risk factors, such as childhood abuse, and proximal risk factors, such as current stressors, for the occurrence of NSSI. Recent studies have demonstrated that the number of life events experienced in the past 6 months can predict the likelihood of an individual engaging in NSSI for the first time in the subsequent year (Kaess et al., 2020). Life events can directly predict NSSI and influence it through other factors (Mo et al., 2019). Specifically, interpersonal life events, such as those involving family or peer groups, have been identified as proximal risk factors for the onset of NSSI (Horvath et al., 2020). These findings highlight the critical role of accumulated stressors and interpersonal challenges in the development of NSSI, emphasizing the need for targeted interventions to mitigate the impact of such events. College students with left-behind experiences may lack appropriate coping strategies for dealing with life events, potentially resorting to negative coping mechanisms such as NSSI to manage stress. This vulnerability may stem from early parent-child separation or reliance on grandparental caregiving (Liu et al., 2021). Therefore, this study hypothesized (H1) that life events are significant predictors of NSSI among college students with left-behind experiences.

Psychotic-like experiences (PLEs) may mediate the relationship between life events and NSSI. PLEs are symptoms that resemble those of clinical psychiatric syndromes, characterized by strange, unusual, or unreal perceptions and thoughts (Wang et al., 2024). These experiences are relatively common in the general population and can persist due to various factors, potentially evolving into mental disorders. PLEs are considered critical early signs of psychiatric disorders (Lindgren et al., 2022). The integrated socio-developmental cognitive model of mental disorders proposes that genetic factors and adverse childhood experiences significantly influence the development of the dopamine system, thereby increasing its sensitivity. Concurrently, stress is exacerbated by irrational cognitive patterns, thereby perpetuating a vicious cycle. Specifically, stress induces dysregulation of dopamine, which in turn generates additional stress, further promoting the release of dopamine and reinforcing paranoid cognitive patterns (Howes and Murray, 2014). Notably, dopamine dysregulation is a critical factor that directly contributes to the onset of mental disorders (Tamminga, 2022). Therefore, life events play an important role in the occurrence of PLEs. Network analysis has found that, recent stressful life events still show a longitudinal association with psychotic symptoms after controlling for long-term social adversity (such as childhood trauma) (Betz et al., 2020). A 2-year prospective study identified stressful life events as a significant risk factor for the recurrence of mental disorders (Colizzi et al., 2023). Hence, it can be concluded that life events can predict PLEs. There is also a connection between PLEs and NSSI. There is evidence suggesting that many mental disorders and NSSI share common neurobiological characteristics, particularly in terms of altered immune-inflammatory responses (Serafini et al., 2020). Individuals with PLEs have a 3-fold higher risk of engaging in NSSI compared to those without PLEs (Honings et al., 2016). The prevalence of PLEs is high in individuals with mental disorders, with a self-injury prevalence rate of 32.6% (Lorentzen et al., 2022). This indicates that the more frequent the PLEs, the more likely NSSI is to occur (Koyanagi and Stickley, 2015). In high-risk adolescents, PLEs are associated with a higher frequency of NSSI, and can be used as an indicator to predict NSSI, including the severity of the behavior (Mylona et al., 2022). Therefore, this study hypothesized (H2) that PLEs mediate the relationship between life events and NSSI among college students with left-behind experiences.

**Figure 1:**
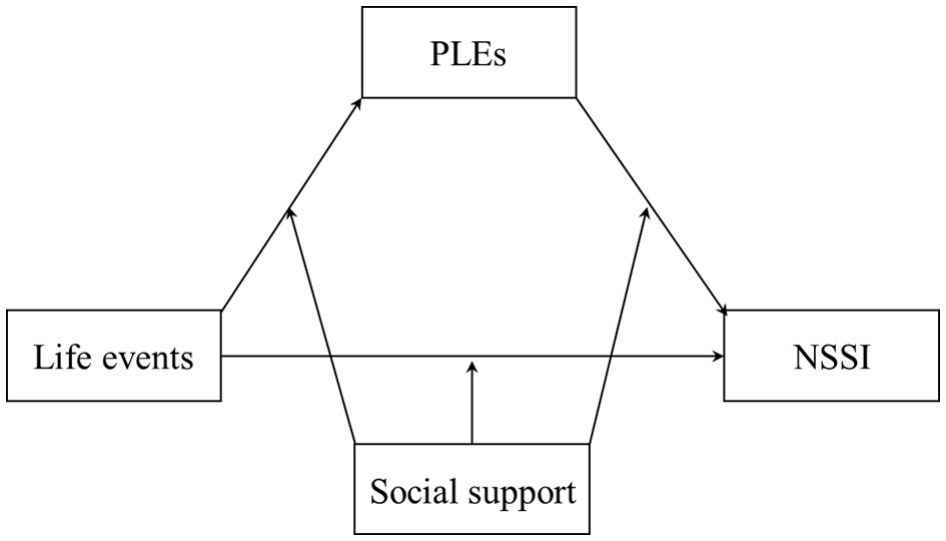
Moderated mediation model hypothesis in the relationship between life events and NSSI.

Social support may play a moderating role in the relationship between life events and NSSI. Social support refers to an individual's perceived or actual experience of being cared for, nurtured, and recognized, or being part of a reciprocal support network (Wills, 1991). The triadic reciprocal causation theory suggests that an individual's psychological and behavioral outcomes are influenced not only by personal factors but also by environmental factors and their interaction (Bandura, 1986). Social support, as an important environmental factor, can influence an individual's psychological and behavioral responses. The stress buffering model suggests that when individuals are in a state of stress, social support can alleviate the impact of stressful events on them, thereby having a positive effect (Cohen and Wills, 1985). In other words, social support can reduce the impact of life events on an individual, improve their psychological wellbeing, and decrease the occurrence of PLEs (Ered et al., 2017) and NSSI (Zhang et al., 2024). Individuals with poor social support are more likely to experience PLEs, which in turn heightens the risk of engaging in NSSI (Saha et al., 2012; Wang and Liu, 2023). Therefore, this study hypothesized (H3) that, among college students with left-behind experiences, social support may play a moderating role in the pathways from life events to NSSI, life events to PLEs, and PLEs to NSSI. The moderated mediation model constructed in this study is shown in Figure 1.

## 2 Methods

### 2.1 Participants

This study included university students from five universities in Hunan and Guangdong provinces. A total of 7,577 questionnaires were collected. Although there was considerable variation in the time taken to complete each item, the overall average response time per item did not fall below 2 s. Therefore, based on the criterion proposed by DeSimone et al. (2015), questionnaires with a total response time of < 534 s were considered invalid and excluded from the analysis, resulting in 5,754 valid responses. In accordance with (Zhang, 2006) definition, individuals were identified as having left-behind experience if they had been separated from one or both parents for more than six consecutive months at any time prior to college. In this study, participants were asked whether their father or mother had worked away from home for over 6 months, which served as the operational criterion for identifying left-behind experience. A total of 2,772 students with left-behind experiences were selected for further analysis, with an average age of 19.214 ± 1.398 years. Among them, 891 were males and 1,881 were females; 1,079 were first-year students, 697 were second-year students, 605 were third-year students, 350 were fourth-year students, and 41 were fifth-year students.

### 2.2 Measures

#### 2.2.1 Adolescent Self-Rating Life Events Checklist (ASLEC)

The Adolescent Self-Rating Life Events Check List (ASLEC), developed by Liu et al. (1997), was used in this study to assess life events over the past 3 months. The scale consists of 27 items covering six factors: interpersonal relationships, academic stress, punishment, loss, health adaptation, and other factors. Participants were asked to report whether they or their families had experienced any of the events listed in the scale over the past 3 months. If an event did not occur, it was scored as 0; if it did occur, participants were required to rate the impact of the event on a 5-point scale: 1 = “no impact,” 2 = “mild,” 3 = “moderate,” 4 = “severe,” and 5 = “extremely severe.” The total score is obtained by summing all item responses, with higher scores reflecting a greater perceived impact of life events on the individual. The Cronbach's α for the ASLEC in the current study was 0.964.

#### 2.2.2 Deliberate Self-Harm Inventory (DSHI)

The brief version of the Deliberate Self-Harm Inventory (DSHI) developed by Gratz (2001) was employed to assess individuals' NSSI behaviors over the past 6 months. The questionnaire included the following instruction: “Have you ever, without intending to kill yourself, deliberately (i.e., not accidentally) engaged in any of the following behaviors? Below are some behaviors through which you may have harmed yourself in the past 6 months. Please read them carefully and select the items that best describe your behaviors.” This ensured that the assessment focused specifically on NSSI, in accordance with its standard definition. The inventory consists of 9 items (e.g., “stabbing the skin with a sharp object”) and uses a 6-point scale (0–5), where 0 indicates “none” and 5 indicates “5 or more times.” The scale has been shown to have good reliability and validity among Chinese student populations (Wang and Liu, 2023). The total score of the DSHI is calculated by summing all item responses, with possible scores ranging from 0 to 45. A score of 0 indicates the absence of NSSI, while any non-zero score reflects the presence of such behavior. Higher scores represent greater severity of NSSI. In the present study, the DSHI demonstrated excellent internal consistency, with a Cronbach's α of 0.908.

#### 2.2.3 Community Assessment of Psychic Experiences (CAPE)

The positive items from the Community Assessment of Psychic Experiences (CAPE) (Mark and Toulopoulou, 2017) were selected to measure PLEs in this study. The scale includes 8 positive items assessing PLEs relates to delusional experiences (e.g., persecutory delusions, thought withdrawal, thought insertion, thought broadcasting, and feelings of control) and hallucinatory experiences (e.g., visual and auditory hallucinations). Participants were asked to rate the frequency of these experiences on a 4-point scale, ranging from 1 (“never”) to 4 (“almost always”). The total score is obtained by summing all item responses, with higher scores indicating more frequent PLEs experienced by the individual. The 8 items from the CAPE have been demonstrated good reliability and validity in the Chinese university students (Zuo et al., 2020). The Cronbach's α for the items in the current study was 0.919.

#### 2.2.4 Perceived Social Support Scale (PSSS)

The Perceived Social Support Scale (PSSS), developed by Zimet et al. (1988) and revised by Huang et al. (1996), is a social support scale that emphasizes individuals' self-perception and understanding of their social support. The scale consists of 12 items, which measure the extent to which individuals perceive support from various sources, such as family, friends, and others. The total score is obtained by summing all item responses, with higher scores reflecting a greater level of perceived social support. The scale has demonstrated good reliability and validity (Chen et al., 2016). The Cronbach's α for the PSSS in the current study was 0.952.

### 2.3 Data analysis

In this study, statistical analyses were conducted using SPSS 25.0 and the PROCESS macro. First, Harman's single-factor test was performed to assess potential common method bias. Subsequently, descriptive statistics were computed, and the reliability of each scale used in the study was evaluated using Cronbach's α coefficient. The prevalence of non-suicidal self-injury (NSSI) among university students was then analyzed. Next, correlation coefficients were calculated to examine the associations between the variables. Finally, the PROCESS macro (Model 59) was employed to investigate the relationships among life events, social support, PLEs, and NSSI.

### 2.4 Multicollinearity and common method variance

Exploratory factor analysis was used to test for possible common method variance by integrating all questionnaire items. The results showed seven factors with eigenvalues >1. The first factor accounted for 26.791%, which was < 40% (Tang and Wen, 2020), no serious common method variance in the data of this study.

## 3 Results

### 3.1 Prevalence of PLEs and NSSI among university students

In the present sample, 62.12% (*n* = 1722) students were reported to have at least one PLE over the past month. The prevalence of PLEs was 62.29% (*n* = 555) among male students, and 62.04% (*n* = 1,167) among female students, There were no significant gender differences in the prevalence of PLEs (χ^2^ = 0.016, *p* > 0.05). Transient PLEs are not pathological, only a small proportion of individuals have frequent PLEs (Sun et al., 2015), Students were categorized as having high frequent PLEs if they selected “often” or “nearly always” on one or more items in this study (Wang et al., 2022). The results showed that 18.51% *(n* = 513) of students reported frequent PLEs, meanwhile, 21.21% (*n* = 189) of male students and 17.22% (*n* = 324) of female students reported such experiences. A significant gender difference was found in the prevalence of frequent PLEs (χ^2^ = 6.373, *p* < 0.05).

The results also showed that 10.55% of participants (*n* = 607) reported at least one instance of NSSI, with a prevalence of 9.45% among males (*n* = 186) and 11.12% among females (*n* = 421). Chi-square tests revealed a significant gender difference in NSSI occurrence (χ^2^ = 3.858, *p* < 0.05), with females being more likely to engage in NSSI. Additionally, chi-square tests indicated a significant difference in NSSI occurrence based on whether participants had left-behind experiences (χ^2^ = 5.210, *p* < 0.05), with students who had left-behind experiences being more likely to engage in NSSI.

### 3.2 Comparison of variables between university students with and without left-behind experiences and gender differences in left-behind experiences

The results, as shown in Table 1, indicated significant differences between university students with and without left-behind experiences regarding age, life events, PLEs, and social support, with a marginally significant difference in NSSI. Additionally, there was a significant gender difference in left-behind experiences (χ^2^ = 10.249, *p* < 0.001). Among females, 49.70% had left-behind experiences, while among males, 45.25% had left-behind experiences, indicating that females are more likely to have experienced being left behind.

**Table 1:** Comparison of variables between university students with and without left-behind experiences.

**Variables**	**With left-behind (*n* = 2,772)**	**Without left-behind (*n* = 2,982)**	***t***
	***M** ±**SD***	***M** ±**SD***	
Age	19.214 ± 1.398	19.121 ± 1.372	2.528^*^
Life events	20.656 ± 18.501	16.103 ± 16.609	9.834^***^
PLEs	2.969 ± 4.049	2.440 ± 3.679	5.185^***^
Social support	60.864 ± 12.672	63.995 ± 12.846	−9.297^***^
NSSI	0.635 ± 3.118	0.493 ± 2.455	1.921^+^

PLEs, psychotic-like experiences; NSSI, non-suicidal self-injury;

+ *p* < 0.1,

* *p* < 0.05,

*** *p* < 0.001.

### 3.3 Descriptive statistics and correlation analysis of variables among university students with left-behind experiences

Table 2 reports the means, standard deviations, and Pearson correlation coefficients for the variables involved in this study. Life events were positively correlated with PLEs (*r* = 0.348, *p* < 0.001) and NSSI (*r* = 0.194, *p* < 0.001), and negatively correlated with social support (*r* = −0.218, *p* < 0.001). PLEs were negatively correlated with social support (*r* = −0.236, *p* < 0.001) and positively correlated with NSSI (*r* = 0.246, *p* < 0.001). Social support was negatively correlated with NSSI (*r* = −0.131, *p* < 0.001).

**Table 2:** Descriptive statistics and correlation analysis (*n* = 2,772).

**Variables**	***M ±SD***	**1**	**2**	**3**	**4**
1 Life events	20.656 ± 18.501	1			
2 PLEs	2.969 ± 4.069	0.348^***^	1		
3 Social support	60.864 ± 12.672	−0.218^***^	−0.236^***^	1	
4 NSSI	0.635 ± 3.118	0.194^***^	0.246^***^	−0.131^***^	1

*** *p* < 0.001.

This study used Model 59 to analyze the standardized variables, while controlling for gender and age differences, to test a moderated mediation model. The model examined whether social support moderated the relationship between life events, PLEs, and NSSI.

Social support significantly moderated all three paths in the proposed model. Specifically, as shown in Table 3, life events significantly predicted PLEs (β = 0.229, *p* < 0.001), social support significantly predicted PLEs (β = −0.171, *p* < 0.001), and the interaction between life events and social support also significantly predicted PLEs (β = −0.070, *p* < 0.001). Therefore, social support moderated the initial part of the mediating effect of life events on PLEs. Similarly, PLEs significantly predicted NSSI (β = 0.180, *p* < 0.001), and the interaction between PLEs and social support significantly predicted NSSI (β = −0.050, *p* < 0.001). Therefore, social support moderated the latter part of the mediating effect of life events on PLEs. In the direct path, life events significantly predicted NSSI (β = 0.106, *p* < 0.001), and the interaction between life events and social support significantly predicted NSSI (β = −0.043, *p* < 0.001). Thus, social support also played a moderating role in the direct effect of life events on NSSI.

**Table 3:** Moderated mediation model (*n* = 2,772).

**Predictors**	**PLEs**			**NSSI**		
	β	***t***	**95% CI**	β	***t***	**95% CI**
Gender	−0.150	−3.978^***^	−0.224, −0.076	0.073	1.845	−0.005, 0.150
Age	−0.015	−1.188	−0.040, 0.010	−0.001	−0.013	−0.026, 0.026
Life events	0.299	16.471^***^	0.263, 0.334	0.106	5.359^***^	0.067, 0.145
Social support	−0.171	−9.519^***^	−0.206, −0.136	−0.065	−3.393^***^	−0.102, −0.027
Life events × social support	−0.070	−3.961^***^	−0.105, −0.036	−0.043	−2.199^*^	−0.081, −0.005
PLEs				0.180	8.934^***^	0.140, 0.219
PLEs × social support				−0.050	−2.798^**^	−0.085, −0.015
*R* ^2^		0.157			0.085	
*F*		103.609^***^			36.435^***^	

The model variables were all standardized; β, Standardized coefficient; *CI*, confidence interval.

* *p* < 0.05,

** *p* < 0.01,

*** *p* < 0.001.

**Figure 2:**
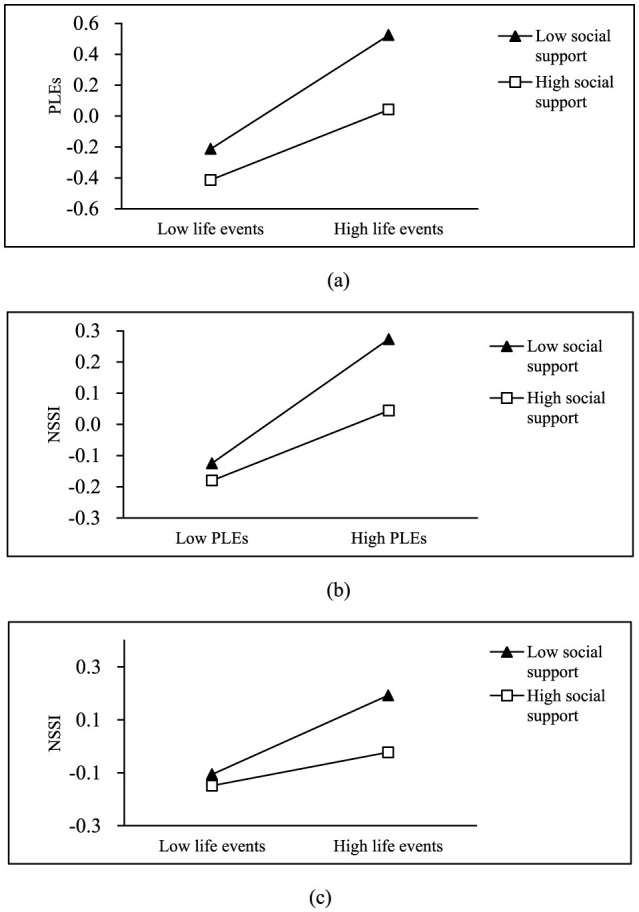
The moderating role of social support in the model. **(a)** Social support moderates the effect of life events on PLEs. **(b)** Social support moderates the effect of PLEs on NSSI. **(c)** Social support moderates the effect of life events on NSSI.

To further explore the interaction effects between variables, simple effects analysis was conducted to examine the moderating role of social support. The results indicated that as the level of social support increased, the predictive effect of life events on PLEs became weaker (β = 0.370, *t* = 15.900, *p* < 0.001; β = 0.228, *t* = 8.337, *p* < 0.001, as shown in Figure 2a), the predictive effect of PLEs on NSSI became weaker (β = 0.230, *t* = 9.353, *p* < 0.001; β = 0.130, *t* = 4.444, *p* < 0.001, as shown in Figure 2b), and the predictive effect of life events on NSSI also became weaker (β = 0.149, *t* = 5.750, *p* < 0.001; β = 0.063, *t* = 2.153, *p* < 0.001, as shown in Figure 2c). This also suggests that social support serves a buffering function.

Table 4 shows a comparison of the mediation effects under different moderation conditions (*M – 1 SD*, 0, *M* + *1 SD*). Since the confidence intervals do not include 0, it indicates that the mediation effects differ under these conditions, confirming that the mediation path is indeed moderated.

**Table 4:** Comparison of mediating effects under different moderating conditions.

**Effect 1**	**Effect 2**	**Difference**	**Boot SE**	**Boot LLCI, Boot ULCI**
0.054	0.085	−0.031	0.020	−0.073, 0.003
0.030	0.085	−0.055	0.032	−0.122, 0.002
0.030	0.054	−0.024	0.012	−0.049, −0.001

Boot SE, bootstrap standard error; Boot LLCI, bootstrap lower limit confidence interval; Boot ULCI, bootstrap upper limit confidence interval.

## 4 Discussion

Life events are risk factors for NSSI, which is consistent with previous research (Zhang et al., 2024), supporting the hypothesis H1. According to (Nock, 2009) Integrated Theoretical Model, one primary function of NSSI is to reduce negative emotions. Since life events are a major source of such emotions, individuals may turn to NSSI as an effective and immediate coping strategy to alleviate the distress caused by these events. As university students often lack life experience and possess limited coping strategies, their ways of handling life events tend to be less effective. This increases their vulnerability to negative emotions and makes them more likely to engage in NSSI as a means of emotional regulation.

University students with left-behind experience tend to exhibit more psychological health problems. According to life course theory (Elder, 1975), an individual's life is embedded within a broader developmental trajectory, and the events and transitions experienced during childhood, adulthood, and old age can all have significant and lasting impacts on the individual. Meanwhile, Thompson (2019) proposed that children can acquire emotion regulation skills by observing their parents, and the stability of the parent-child relationship can also influence the individual's emotional development. University students with left-behind experience are affected psychologically by parental absence. Additionally, childhood separation from their parents deprives these individuals of opportunities to acquire adaptive skills through parental guidance. Furthermore, reliance on remote communication often limits interactions between children and their parents to a narrow range of topics, such as academic performance, which may lead to the neglect of the children's emotional needs. Collectively, these factors can impede the development of effective emotion regulation capacities. Consequently, when faced with life events, these individuals are more likely to experience heightened negative emotions. Their limited adaptive emotion regulation skills, in turn, increase the risk of engaging in NSSI.

PLEs were shown to partially mediate the relationship between life events and NSSI, implying that life events may predict NSSI through the influence of PLEs, thereby confirming hypothesis H2. The integrated social development cognitive model of mental disorders suggests that the development of mental illness is linked to childhood adversities and life events faced by individuals (Howes and Murray, 2014). For university students with left-behind experiences, negative early experiences can affect the dopamine system, thereby increasing their perception of stress from life events (Iqbal et al., 2022). This can lead to more erroneous attributions, with the resulting paranoia and hallucinations further increasing stress, which in turn enhances the individual's PLEs. Additionally, paranoia serves as a barrier to cognitive reappraisal in emotional regulation. When individuals are unable to use appropriate emotional regulation strategies, they may turn to quick and effective NSSI as a means of self-adjustment.

In addition to examining the mediating role of PLEs, this study further explored the moderating role of social support in this process. The results demonstrated that social support moderated the first-stage path, the second-stage path, as well as the direct path through which life events influence NSSI via PLEs, thereby supporting Hypothesis H3. According to the stress-buffering model of social support (Cohen and Wills, 1985), social support serves as a key psychological resource that mitigates the adverse effects of stressful life events. Specifically, when individuals encounter PLEs, social support may help them recognize the unreality or extremity of such experiences, thereby promoting timely psychological adjustment. Moreover, it can offer individuals more effective problem-solving strategies and emotional regulation techniques, enabling them to experience emotional warmth and adopt more adaptive coping strategies, which in turn may alleviate maladaptive PLEs and reduce the likelihood of NSSI. Conversely, a lack of sufficient social support may exacerbate the psychological burden of life events, heighten the occurrence of PLEs, and lead to social withdrawal, thereby increasing the risk of NSSI.

Notably, the strength of these moderated mediation effects may vary across groups. Students with left-behind experience often exhibit heightened interpersonal sensitivity—such as feelings of inferiority, uneasiness, discomfort, restraint, and negative expectations (Liu et al., 2021)—which may predispose them to perceive life events as more stressful or overwhelming. These psychological tendencies not only amplify the subjective impact of external stressors but also increase their vulnerability to psychological distress when facing adverse circumstances. As a result, they may be more susceptible to maladaptive coping strategies such as NSSI when confronted with stressful life events. In contrast, non-left-behind students may perceive fewer life stressors and generally report higher levels of social support. Consequently, the buffering effect of social support on the relationship between life events and NSSI may be less pronounced—or even statistically non-significant—in this group, possibly due to lower baseline stress levels or more effective coping resources.

## 5 Significance and limitations

This study holds important implications for the prevention and intervention of NSSI among university students, particularly those with left-behind experiences, who may face elevated psychological challenges due to cumulative stressors. It is essential to recognize the role of life events in shaping students' wellbeing and to monitor significant stressors they encounter in both academic and personal contexts. Building strong teacher-student relationships and being attentive to potential signs of PLEs, such as auditory or visual hallucinations, can facilitate timely support. Moreover, fostering robust social support systems—especially for students with limited support—through group activities and peer engagement can enhance resilience. For students at risk of NSSI, teaching emotional regulation strategies, such as cognitive reappraisal, alongside social support enhancement, may help reduce PLEs and prevent further NSSI.

In addition, this study has certain limitations. Firstly, this study relied on self-reported questionnaires, with data obtained solely through self-reports. Since PLEs may not be fully understood and NSSI is often stigmatized, there may be issues related to the reliability of the data. Future studies could incorporate data from multiple sources for a more comprehensive analysis. Secondly, this study utilized cross-sectional data, which is essentially correlational in nature. Future research could use longitudinal data to explore the causal relationships between the variables. Finally, perceived social support refers to the support individuals feel they receive, which may differ from actual social support. This study did not delve into objective social support, and future research could explore the role of objective social support in this process.

## 6 Conclusion

(1) In the group of university students with left-behind experiences, life events can positively predict NSSI.

(2) PLEs mediate the relationship between life events and NSSI.

(3) Social support plays a moderating role in this mediation model, as it can reduce the direct impact of life events on NSSI, as well as weaken the indirect effects.

## Data Availability

The original contributions presented in the study are included in the article/supplementary material, further inquiries can be directed to the corresponding authors.
